# Association Between a State-Level Fat Tax and Fast Food Purchases

**DOI:** 10.1001/jamanetworkopen.2023.37983

**Published:** 2023-10-16

**Authors:** Sumit Agarwal, Pulak Ghosh, Changwei Zhan

**Affiliations:** 1 NUS Business School, National University of Singapore (NUS), Singapore; 2 Department of Decision Sciences and Centre for Public Policy, Indian Institute of Management Bangalore, Bangalore, India; 3 NUS Business School and Institute of Real Estate and Urban Studies, NUS, Singapore

## Abstract

**Importance:**

India faces an increasing obesity problem, including in the Indian state of Kerala in which the fat tax was implemented but was nullified 11 months later. A fat tax, defined as a tax on unhealthy foods, may be associated with changes in food purchases and outcomes for multiple diet-related diseases.

**Objective:**

To investigate the association between the state-level fat tax and fast food purchases in Kerala, India.

**Design, Setting, and Participants:**

This cohort study analyzed a large-scale credit and debit card transaction data set and aggregated this sample at the account-year-month level of fast food purchases in Kerala state and 9 major cities in other Indian states (Ahmedabad, Bangalore, Bhubaneswar, Chennai, Delhi, Gurgaon, Kolkata, Mumbai, and Surat). Purchase records were obtained for January 1, 2016, to December 31, 2017. The association between the fat tax and fast food purchases was examined using the difference-in-differences method. This analysis was initiated on December 1, 2022.

**Exposures:**

The exposure was the fat tax. Kerala was the exposed group, and 9 major Indian cities were the control group.

**Main Outcomes and Measures:**

The main outcome was the fast food purchase ratio, defined as the proportion of fast food purchases of the total food purchases. Changes in the fast food purchase ratio were estimated in Kerala across the sample period and then compared with 9 major cities.

**Results:**

The sample for analysis included 238 015 credit and debit card accounts, of which 36.7% were in Kerala and 63.3% were in 9 major cities. The cardholders included 191 603 males (80.5%) with a mean (SD) age of 36.6 (12.8) years. During the fat tax implementation (August 2016-June 2017), Kerala’s fast food purchase ratio decreased by 3.9 percentage points (β [SE], −0.039 [0.002]; 95% CI, −0.042 to −0.036), compared with 9 major cities. After the fat tax was nullified, the fast food purchase ratio reduced by 5.6 percentage points (γ [SE], −0.056 [0.002]; 95% CI, −0.059 to −0.052) compared with 9 major cities and using the pretax period as the benchmark.

**Conclusions and Relevance:**

Results of this cohort study suggest that the Kerala fat tax was associated with fewer fast food purchases. Food tax policies need to have an elaborate design, and related issues, such as social inequality, nutritional deficiency, and political concerns, need to be evaluated in future studies.

## Key Points

**Question:**

Was the fat tax in Kerala, India, associated with fewer fast food purchases?

**Findings:**

In this cohort study of 238 015 credit and debit card accounts, the Kerala fat tax was associated with a 3.9–percentage point decrease in the fast food purchase ratio, defined as the proportion of fast food purchases of the total food purchases, among cardholders. After the fat tax was nullified, the fast food purchase ratio was significantly reduced by 5.6 percentage points compared with the pretax period.

**Meaning:**

Findings of this study suggest that the fat tax was associated with fewer fast food purchases.

## Teaser

This cohort study analyzes credit and debit card transactions for foods purchased at branded restaurants or fast food chains before and after implementation of a fat tax in Kerala state and 9 major cities in India.

## Introduction

Globally, more than 1 billion people have obesity, according to the World Health Organization.^1^ Obesity is associated with multiple noncommunicable diseases (NCDs), such as type 2 diabetes, cardiovascular disease, and certain types of cancer. This worldwide epidemic of high body mass index was responsible for more than 5 million deaths, as estimated by the 2019 Global Burden of Disease study.^2^ Even though the definitions of obesity and body mass index vary across countries and regions, many governments have implemented various public policies to tackle the prevalence of obesity and overweight. The policy tools include sugar tax, nutrition labeling, advertising restrictions, and public awareness campaigns. Taxing unhealthy food or subsidizing healthy food are fiscal tools commonly adopted by governments. For example, in 2011, Finland introduced taxes on sweets and sugary drinks, Denmark enacted the world’s first *fat tax* (defined as a tax on unhealthy foods) on saturated fat but repealed it 15 months later, and Hungary levied higher taxes on unhealthy foods high in fat.^3,4^ Other countries and regions, such as France, Mexico, UK, and various US states, also included sugar-sweetened food and saturated fat or generally defined junk food in their taxation lists.^3,5^

Previous research mainly evaluated the sugar tax’s effectiveness since it is a more prevalent taxation mode. These studies found that sugar taxes were associated with lower consumption of sugar-sweetened food and beverages.^6,7,8,9,10,11^ The present study examined India’s fat tax, which loosely targeted junk food sold by branded restaurants. Related work evaluated socioeconomic data and model consumption as a response to fat taxes.^12,13,14,15,16,17^ Other studies, based on experiments, fieldwork, or empirical analysis, analyzed the outcomes of fat taxes.^18,19,20,21,22,23^ Generally, there is still insufficient empirical evidence to demonstrate fat taxes’ influence and welfare gains. In addition, poorly designed fat tax policies may be associated with issues such as substituting taxed food with unhealthier food, increasing the burden on groups with lower socioeconomic status, and political lobbying.^3,19,24,25,26,27^

India faces an increasing obesity problem. According to a national survey in 2015 to 2016, the percentage of the country’s population with overweight or obesity was 18.9% in males and 20.7% in females.^28^ Kerala, a southwestern state with wealth and obesity rates that are in the upper tier in India, reached overweight rates of 28.5% in males and 32.4% in females in the same national survey.^28^

In the first budget of July 2016, the Minister of Finance of Kerala introduced the fat tax to the public. The fat tax was an indirect tax of 14.5% levied on burgers, pizzas, tacos, doughnuts, sandwiches, pasta, and bread fillings sold by restaurants with a brand name or registered trademark. This taxation was based on the type of restaurant instead of ingredients or specific food products, which were the usual basis in other countries. As stated by the Kerala government, there were 2 purposes for introducing the fat tax. First, the tax was a part of the government’s efforts to address the increasing prevalence of NCDs. Second, the revenue generated from the tax was intended to be used for public health initiatives, including promoting healthy eating habits and raising awareness about the risks of consuming foods with high content of unhealthy fats.

The Kerala fat tax went into effect in August 2016. It existed for 11 months until June 2017. In 2017, the Indian federal government launched a new taxation system: the nationwide Goods and Services Tax (GST). The GST replaced all state-level indirect taxes on goods and services with federal uniformed tax rates. Therefore, the Kerala fat tax expired in July 2017. Although the Kerala fat tax lasted for less than 1 year, it may still be associated with consumers’ eating habits during and after the validity period. Policy details of this fat tax are discussed in eAppendices 1 and 2 in Supplement 1. In this cohort study we aimed to investigate the association between the state-level fat tax and fast food purchases in Kerala, India.

## Methods

The NUS Business School, National University of Singapore Faculty Ethics Review Committee deemed this study exempt from ethics review and waived the informed consent requirement because the study used existing, deidentified archival data and presented low risk. We followed the Strengthening the Reporting of Observational Studies in Epidemiology (STROBE) reporting guideline.

The analysis was based on a large-scale card transaction data set from the ICICI Bank, a top-tier private bank in India. In the original data set, each observation denoted a single transaction through either a credit card or a debit card. The original data set was composed of a randomly selected sample from all ICICI Bank card transactions. Basic information obtained included transaction date, amount (in rupees [€]), card type (credit or debit card), unique card identifier (account ID), and 4-digit merchant category code (MCC). Considering that the Kerala fat tax was implemented in August 2016 and nullified in July 2017, we restricted the sample period to 2016 to 2017. Spatially, we included card transactions between January 1, 2016, and December 31, 2017, in Kerala, including all cities and other areas in the state (representing the exposed group). Additionally, we collected transactions in 9 major cities in other Indian states: Ahmedabad, Bangalore, Bhubaneswar, Chennai, Delhi, Gurgaon, Kolkata, Mumbai, and Surat (representing the control group). Due to limited data availability, we did not have card transactions for locations beyond Kerala and the 9 major cities.

We also combined card transactions and cardholder characteristics, which the bank provided, using the unique cardholder identifier. These characteristics depicted each cardholder’s basic demographic profile, including age, sex, profession, and marital status. Using customer attributes, we could test the heterogeneous responses to the Kerala fat tax; that is, whether customers with distinct demographics responded to the fat tax differently.

**Figure 1.**
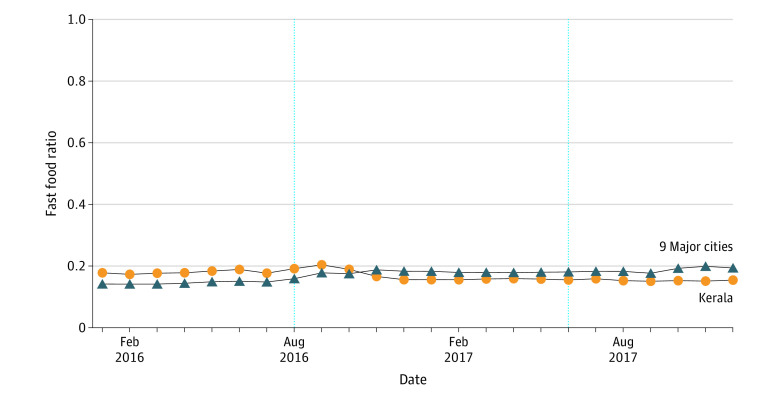
Patterns of Fast Food Purchase Ratio

Using the MCC, we could distinguish the amount the cardholder paid to which merchant category for each transaction. In this study, we identified 2 categories of merchants: fast food restaurants (MCC = 5814) and eating places and restaurants (hereafter referred to as *other food*; MCC = 5812). We then aggregated the sample at the account-year-month level, including 238 015 accounts and 24 months in 2016 and 2017. Briefly, we derived how much the cardholder consumed at fast food (*FastFood* in formula) and other food (*OtherFood* in formula) restaurants during a specific month. The outcome of this baseline analysis was the fast food purchase ratio (FastFoodRatio in formula), which was defined as the proportion of fast food purchases of the total food purchases and was calculated as follows: *FastFood* / (*FastFood* + *OtherFood*). We used the fast food purchase ratio rather than the absolute fast food purchases to mitigate the concerns of confounding factors, such as local fiscal policy changes, in Kerala and 9 major cities. The outcome FastFoodRatio ranged from 0 to 1, and we assigned to it the missing value if no food purchase was recorded in the raw data. Hence, we could see the changing patterns in fast food purchase proportions in Kerala vs 9 major cities (Figure 1).

### Statistical Analysis

The research design was straightforward, using the difference-in-differences (DID) method. We started by estimating the association between the fat tax and fast food purchases using the following equation:



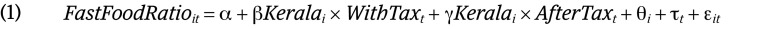



As this equation shows, the dependent variable was the fast food purchase ratio of account *i* in month *t* (January 2016-December 2017). In the DID interaction terms, the dummy variable Kerala*_i_* took the value of 1 to indicate that an account was registered in Kerala and the value of 0 for registration in the 9 major cities. Dummy variable WithTax*_t_* denoted 1 to represent the period with the fat tax (August 2016-June 2017); otherwise, it denoted 0. Dummy variable AfterTax*_t_* denoted 1 to represent the period after the fat tax was canceled (July-December 2017); otherwise, it denoted 0. We assigned the pretax period (January-July 2016) as the benchmark. To control for time-invariant account-specific characteristics and time-varying factors, we included account fixed effects *θ_i_* and year-month fixed effects *τ_t_* in the basic specification. In stricter specifications, we also included more granular city-year fixed effects (with Kerala considered to be a city alongside the other cities) or account-year fixed effects as robustness. The error term was *ϵ_it_*. Robust SEs were clustered at the account level. The estimated β and γ coefficients and the statistical significance (2-sided *P* value) indicated the associations between the fat tax and fast food purchases in Kerala.

Besides the static estimation, we also examined the dynamic responses to the fat tax using the event study strategy, as shown in the following equation:
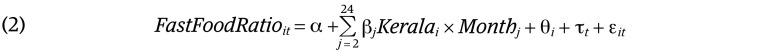
We split the sample into 24 months (January 2016-December 2017). We then generated 24 corresponding dummy variables and assess the interaction of each of them with *Kerala_i_*. We set the first month of the sample (January 2016) as the benchmark period. Therefore, we could estimate the coefficients of 23 interaction terms, and they represented the relative difference in fast food purchase ratio in Kerala compared with 9 major cities in each corresponding month, with January 2016 as the benchmark.

In addition to the baseline analysis, we conducted heterogeneous tests using the cardholder information documented by ICICI Bank. There were 4 individual characteristics, and we compared cardholders by age group (31-90 years vs 18-30 years), sex (female vs male), marital status (married vs single), and profession (public sector, public limited, and government job vs other job). We ran event studies as Equation 2 on different groups to show their dynamic heterogeneous responses.

We conducted additional tests to check the robustness of the baseline results. First, we deleted accounts without any fast food purchases before August 2016. Second, we used an alternative outcome variable called *fast food frequency ratio*, defined as the frequency of fast food purchases as a percentage of total food purchases. Third, for accounts in Kerala, we kept only those in 3 populated regions: Trivandrum Headquarter, Calicut, and Kochi. These robustness tests were conducted using event studies.

Data analysis was performed with Stata, version 15.0 (StataCorp LLC). We initiated the analysis on December 1, 2022.

## Results

The analysis included a sample of 238 015 credit and debit card accounts, of which 36.7% were from Kerala and 63.3% were from 9 major cities. Among the cardholders, 191 603 (80.5%) were males and 46 412 (19.5%) were females, with a mean (SD) age of 36.6 (12.8) years. Additionally, 58.6% were married and 41.4% were single, and 5.0% were employed in the public sector, while 95.0% held other types of jobs. Summary statistics of the full sample, including the exposed group (state of Kerala) and the control group (9 major cities), are provided in the eTable in Supplement 1.

Estimation of the association between the Kerala fat tax and fast food purchases is shown in the Table. During the period of fat tax implementation, the exposed group’s fast food purchase ratio decreased by 3.9 percentage points (β [SE], −0.039 [0.002]; 95% CI, −0.042 to −0.036) compared with the control group (Table). After the Kerala fat tax was replaced by the GST, the exposed group’s fast food purchase ratio decreased by 5.6 percentage points (γ [SE], −0.056 [0.002]; 95% CI, −0.059 to −0.052) compared with the control group and using the pretax period as the benchmark. We controlled for both account fixed effects and year-month fixed effects, and these estimates were significant at the 1% level. When more granular fixed effects were included, the estimated (SE) coefficients of the association were robust during the fat tax period (ranging from −0.031 [0.002; 95% CI, −0.034 to −0.027] to −0.030 [0.002; 95% CI, −0.033 to −0.026]) and after the tax was nullified (ranging from −0.042 [0.002; 95% CI, −0.046 to −0.037] to −0.040 [0.002; 95% CI −0.045 to −0.036]) (Table).

**Table.** Fat Tax and Fast Food Purchase Ratio in Kerala State and 9 Major Cities^a^

Model	Estimate (SE) [95% CI]	*P* value
**Model A: with account FE and year-month FE (n = 1 736 798)**		
Kerala*_i_* × WithTax*_t_*, β	−0.039 (0.002) [−0.042 to −0.036]	<.001
Kerala*_i_* × AfterTax*_t_*, γ	−0.056 (0.002) [−0.059 to −0.052]	<.001
Constant	0.184 (0.000) [0.183 to 0.184]	<.001
**Model B: with account FE, year-month FE, and city-year FE (n = 1 527 157)**		
Kerala*_i_* × WithTax*_t_*, β	−0.031 (0.002) [−0.034 to −0.027]	<.001
Kerala*_i_* × AfterTax*_t_*, γ	−0.042 (0.002) [−0.046 to −0.037]	<.001
Constant	0.181 (0.001) [0.179 to 0.182]	<.001
**Model C: with account-year FE and year-month FE (n = 1 685 841)**		
Kerala*_i_* × WithTax*_t_*, β	−0.030 (0.002) [−0.033 to −0.026]	<.001
Kerala*_i_* × AfterTax*_t_*, γ	−0.040 (0.002) [−0.045 to −0.036]	<.001
Constant	0.180 (0.001) [0.179 to 0.181]	<.001

Abbreviation: FE, fixed effect.

^a^ The exposure group comprised debit and credit card accounts in the state of Kerala denoted by the dummy variable Kerala*_i_*, with *_i_* indicating the account. The control group comprised debit and credit card accounts in 9 major cities in India. The dummy variable WithTax*_t_*, with *_t_* indicating the month, denoted the period during fat tax implementation (August 2016-June 2017), and the dummy variable AfterTax*_t_* denoted the period after the fat tax was nullified (July 2017-December 2017). The pretax period (January 2016-July 2016) was the benchmark. We controlled for different FEs in 3 models (A, B, and C). Observations varied because singleton observations were dropped. Robustness SEs were clustered at the account level.

**Figure 2.**
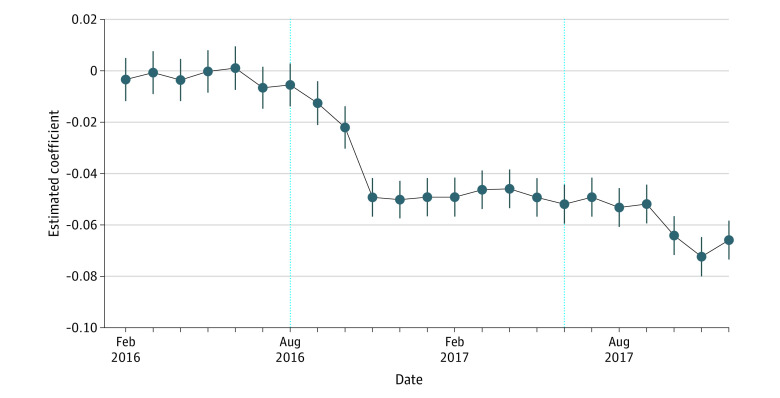
Dynamic Changes in Fast Food Purchase Ratio Account fixed effects and year-month fixed effects were controlled. Robust SEs were clustered at the account level. Error bars represent 95% CIs.

Using an event study design, we tested the dynamic association between the Kerala fat tax and the fast food purchase ratio (Figure 2). During the pretax period, estimated coefficients in each month were undistinguishable from 0, which indicated the comparability between Kerala and 9 major cities and the validity of the DID method. After the fat tax implementation, there were significant differences in the fast food purchase ratio between Kerala and 9 major cities, and the estimations ranged approximately from 2.0 to 6.0 percentage points. At the inception of the fat tax, the Kerala government publicly introduced the taxation plan to the public. When the fat tax was nullified due to the new GST, the downward pattern of the fast food purchase ratio continued even after the cessation of the fat tax (β [SE], −0.039 [0.002], 95% CI, −0.042 to −0.036; γ [SE], −0.056 [0.002], 95% CI, −0.059 to −0.052). One possible reason may be that the cessation of the fat tax was not salient to customers; hence, there was no rebound in fast food purchases after June 2017.

Results of the robustness tests are consistent with the baseline findings and are provided in eFigure 1 in Supplement 1. In all 3 tests, the fast food purchase ratio in Kerala decreased by up to approximately 6 percentage points during the tax period and up to approximately 8 percentage points after the tax was nullified, compared with the control group.

**Figure 3.**
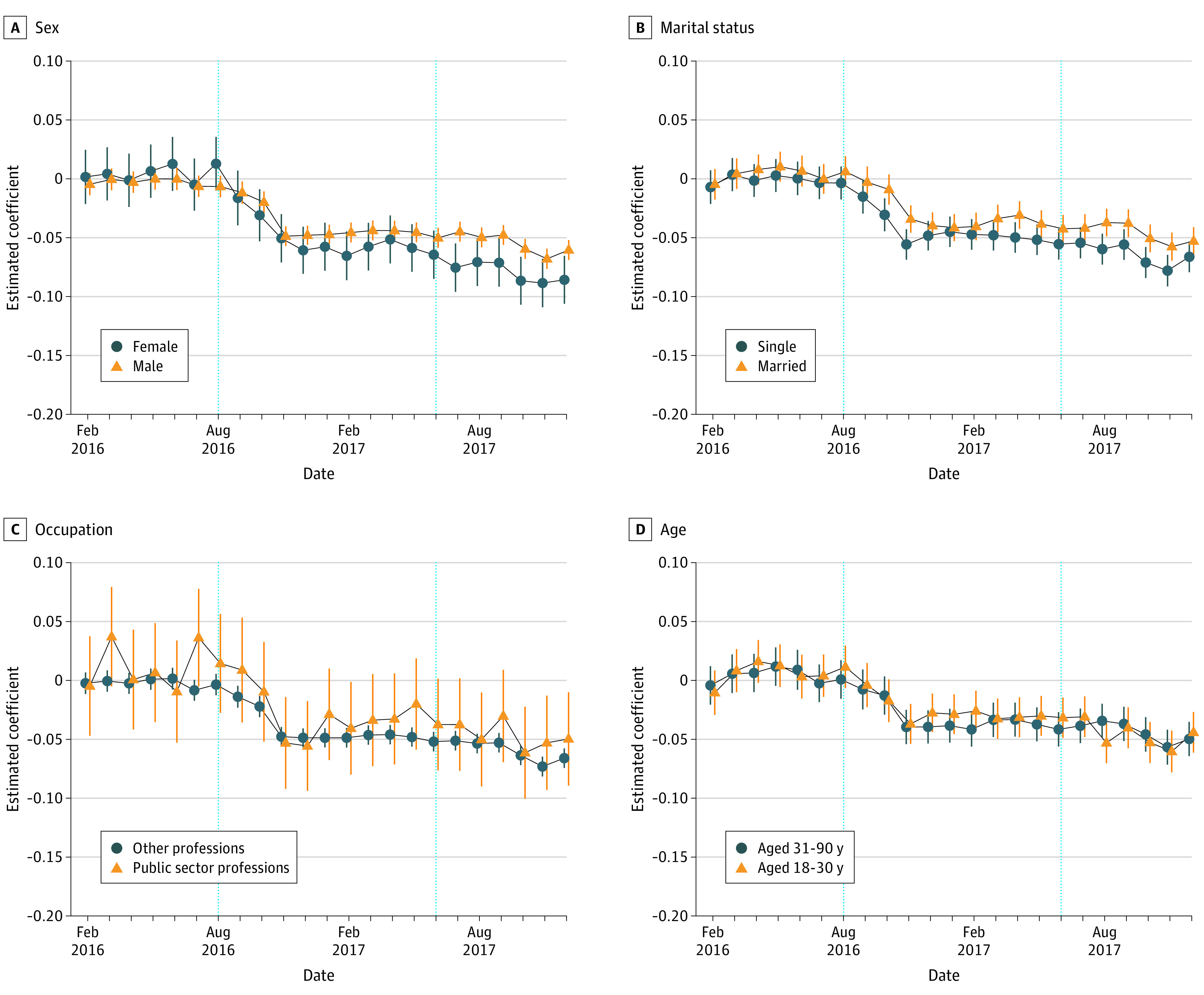
Changes in Fast Food Purchase Ratio Stratified by Sex, Marital Status, Occupation, and Age Account fixed effects and year-month fixed effects were controlled. Robust SEs were clustered at the account level. Error bars represent 95% CIs.

The fat tax was associated with reduction in fast food purchases in Kerala. The follow-up question was, did people’s socioeconomic status affect their response to the fat tax? Figure 3 shows the heterogeneous responses of customers in different subgroups of age, sex, marital status, and profession. Compared with males, female cardholders had a relatively larger decrease in fast food purchases after the fat tax adoption in Kerala vs 9 major cities (males: β [SE], −0.037 [0.002], 95% CI, −0.040 to −0.034 and γ [SE], −0.052 [0.002], 95% CI, −0.055 to −0.048; females: β [SE], −0.051 [0.004], 95% CI, −0.059 to −0.043 and γ [SE], −0.080 [0.005], 95% CI, −0.089 to −0.071). Cardholders who were single had a relatively larger decrease in fast food purchases, compared with cardholders who were married, in Kerala vs 9 major cities (single: β [SE], −0.042 [0.003], 95% CI, −0.047 to −0.037 and γ [SE], −0.062 [0.003]; 95% CI, −0.068 to −0.056; married: β [SE], −0.034 [0.002], 95% CI, −0.038 to −0.029 and γ [SE], −0.049 [0.003], 95% CI, −0.054 to −0.043). Even though the heterogeneities were not statistically significant in most months, they still potentially indicated that single and female cardholders were more price sensitive or concerned about obesity-related health issues. However, we did not find heterogeneous responses to the fat tax between groups stratified by age or profession.

The fat tax was introduced by the then–newly elected communist party, the Left Democratic Front (LDF). The fat tax disproportionately targeted junk foods sold at branded restaurants, most of which were the so-called Western food chains. Therefore, critics viewed LDF’s fat tax as politically motivated. Hence, we conducted a heterogeneous test that compared the responses to the fat tax in pro-LDF districts vs non-LDF districts, using the results of the 2016 Kerala general election. The results are provided in eFigure 2 in Supplement 1. Overall, we found that the decreases in fast food purchases associated with the fat tax in pro-LDF districts (β [SE], −0.039 [0.002], 95% CI, −0.043 to −0.035; γ [SE], −0.055 [0.002], 95% CI, −0.059 to −0.051) were similar in non-LDF districts (β [SE], −0.039 [0.002], 95% CI, −0.044 to −0.035; γ [SE], −0.057 [0.003]; 95% CI, −0.062 to −0.052).

## Discussion

In this cohort study of the association between the Kerala fat tax and fast food purchases, baseline estimations indicated that compared with 9 major cities, in Kerala the fat tax was associated with a 3.9–percentage point decrease in the fast food purchase ratio. After the fat tax was discontinued, the fast food purchase ratio in Kerala was significantly reduced by 5.6 percentage points compared with the pretax period. Moreover, we found differential responses to the fat tax among cardholders with different demographic characteristics, such as sex and marital status, and socioeconomic status.

Obesity and its related diseases are global concerns, and several countries have proposed and enacted policy tools to address the problem, including fat taxes. However, empirical analysis of these policy implementations remains scarce, especially analysis using large-scale transactional data.

### Limitations

This study has limitations. First, because the bank transaction data had limited information, we could identify only merchant categories instead of specific merchants or food products. However, because the fat tax was levied on branded restaurants rather than food items, the identification issue may not be a big concern. Second, due to the nature of archival data, the demographics in the present sample were different from the demographics of the wider Indian population. We mitigated this concern by controling for account-level fixed effects. Third, even though the sample included a large number of credit and debit card accounts, we could not detect changes in fast food purchases through cash and other payment methods due to lack of data.

## Conclusions

This cohort study provided new empirical evidence of the associations between a fat tax and food purchases based on the policy practice of a resource-limited economy (Kerala, India). Compared with past fat tax or sugar tax practices in high-income countries, the fat tax in the Indian state of Kerala was primarily based on taxing fast foods sold at branded restaurants, instead of specific ingredients or food products. Associated with decreased fast food purchases, the fat tax might have health benefits, which are vital to Kerala and other regions in India where obesity, diabetes, and other NCDs are prevalent. Moreover, a food tax policy needs to have an elaborate design, and related issues, such as social inequality, nutritional deficiency, and political concerns, need to be evaluated in future studies.

## Notes

Supplement 1. **eAppendix 1.** Policy Background of the Fat Tax in Kerala **eAppendix 2.** The Fat Tax and Fast Food Purchases **eFigure 1.** Robustness Checks **eFigure 2.** Differential Responses to the Fat Tax by Political Leaning **eTable.** Summary Statistics

Supplement 2.
Data Sharing Statement
