# Supplementary material for: Association Between a State-Level Fat Tax and Fast Food Purchases
Source: JAMA Netw Open. 2023 Oct 16;6(10):e2337983. doi: 10.1001/jamanetworkopen.2023.37983 (PMC10580107; doi:10.1001/jamanetworkopen.2023.37983)
Supplement: Supplement 1. — eAppendix 1. Policy Background of the Fat Tax in Kerala eAppendix 2. The Fat Tax and Fast Food Purchases eFigure 1. Robustness Checks eFigure 2. Differential Responses to the Fat Tax by Political Leaning eTable. Summary Statistics [file jamanetwopen-e2337983-s001.pdf]

Supplemental Online Content

Agarwal S, Ghosh P, Zhan C. Association between a state-level fat tax and fast food purchases in India. *JAMA Netw Open*. 2023;6(10):e2337983. doi:10.1001/jamanetworkopen.2023.37983

eAppendix 1. Policy Background of the Fat Tax in Kerala

eAppendix 2. The Fat Tax and Fast Food Purchases

eFigure 1. Robustness Checks

eFigure 2. Differential Responses to the Fat Tax by Political Leaning

eTable. Summary Statistics

This supplemental material has been provided by the authors to give readers additional information about their work.

© 2023 Agarwal S et al. *JAMA Network Open*

eAppendix 1. Policy background of the fat tax in Kerala

In this section, we detail the policy and institutional background of the fat tax as a supplement to the main text.

The health conditions of Indian people, including those in Kerala, have been a cause for concern in recent years. India is experiencing a rapid epidemiological transition, with Non-Communicable Diseases (NCDs) emerging as a major public health challenge. According to the National Family Health Survey (NFHS-4) conducted in 2015 – 2016, the countrywide percentages of people who are overweight or obese are 18.9% in men and 20.7% in women. While in Kerala, a relatively richer state in India, the obesity and overweight rates reached 28.5% in men and 32.4% in women, as documented by the same survey. In response to the escalating impact of NCDs in the nation, the Indian federal government initiated the National Program for Prevention and Control of Cancer, Diabetes, Cardiovascular Diseases, and Stroke (NPCDCS) in 2010-11. Kerala was among the first states that adopt the NPCDCS program.

In 2016, the Left Democratic Front (LDF), a left-wing communist party, won the general election in Kerala and came to power. The alarming statistics of obesity-related diseases prompted the government of Kerala to take action to address the issue through various policy interventions. The newly elected government initiated a series of policy tools, including the Sustainable Development Goals (SDG) framework. According to the SDG, the government set specific targets to reduce the burden of NCDs. For example, it aimed to curb the rising trend of obesity and reduce premature mortality from NCDs by 5% by 2020 and 10% by 2030.

In the first budget in July 2016, the Minister of Finance of Kerala first introduced the fat tax to the public. The fat tax aimed to reduce the consumption of unhealthy foods, specifically foods high in saturated and trans fat. The tax rate was set as 14.5%, levied on burgers, pizzas, tacos, doughnuts, sandwiches, pasta, and bread-fillings sold by restaurants having a brand name or trade mark registered under the Trade Marks Act, 1999, for example, McDonald's, Domino's, Pizza Hut, etc. The Kerala fat tax is different from other countries' practices by taxing ingredients or high-calorie/high-fat food items. For example, the fat tax in Denmark was levied on saturated fats in food products, it added 16 kroner ($2.7) per kilogram of saturated fats in a product. The Kerala fat tax targeted loosely defined "fast food" sold by branded restaurants. As stated by the government, there were two purposes for introducing the fat tax. First, the tax was also proposed as a part of the Kerala government's efforts to address the rising prevalence of NCDs. Second, The revenue generated from the tax is intended to be used for public health initiatives, including promoting healthy eating habits and raising awareness about the risks of consuming foods high in unhealthy fats. The fat tax was an indirect tax. For example, a 114.5 Rupees purchase of fast food includes 100 Rupees pre-tax price and a 14.5 Rupees fat tax.

© 2023 Agarwal S et al. *JAMA Network Open*

Considering that the fat tax was announced by the Finance Minister of Kerala in his budget speech in July 2016, therefore, the customers were aware of the implementation of the fat tax, or in other words, the fax tax was salient to the public.

The fat tax was officially implemented in August 2016 and met with mixed reactions. Proponents argue that it is necessary to discourage the consumption of unhealthy foods and promote healthier eating habits. However, criticisms and concerns remained. Considering that the government only proposed to levy taxes on branded restaurants, and these restaurants were more of a so-called Western diet, which only accounted for a small part of the food market. Meanwhile, the traditional Indian diet also contains much unhealthy food high in oil. Critics have questioned the effectiveness of the fat tax, especially given that a left-leaning communist government introduced it.

In 2017, the Indian federal government launched a new taxation system, a nationwide Goods and Services Tax (GST). The new GST replaced all state-level indirect taxes on goods and services with federal uniformed tax rates. Therefore, the Kerala fat tax expired in July 2017. Although this relatively short-lived fat tax only lasted for one year, it may still potentially influence consumers' eating habits during and after the validity period. In our study as documented in the main text, we also showed changes in fast food purchases after the fat tax was lifted. There is currently no empirical analysis of the association between the fat tax and food purchases based on big data, and our study fills this gap.

eAppendix 2. The fat tax and fast food purchases

In the main text, we employed the Difference-in-Differenes model and found that the fat tax resulted in a more than 3.0 percentage points decrease in the fast food purchase ratio. The sample for analysis includes 238,015 accounts with 36.7% in Kerala, 63.3% in other cities, 80.5% males, 19.5% females, 58.6% married, 41.4% single, 5.0% public-sector jobs, 95.0% other jobs, and an average (SD) age of 36.6 (12.8). We also showed summary statistics of the full sample the Kerala group, and the control gorup (other nine cities) in the eTable.

In this supplementary section, we would like to conduct additional tests to examine the robustness of the baseline analysis. First, cardholders (customers) who launched a card earlier and those later cardholders might perform differentially in food purchases. Hence, we deleted accounts without any fast food purchases before August 2016 to have more consistent account observations in the sample. Second, in the main text, the dependent variable was set as the transaction amount of fast food as a proportion of all food transaction amounts. Here, we used an alternative outcome variable called "fast food frequency ratio", defined as the frequency of fast food purchases as a percentage of the frequency of total food purchases. Finally, due to data restrictions, besides Kerala, we only have transactions in nine major cities but not in other regions. One may concern that Kerala may not be comparable to those big cities. To mitigate this concern, for accounts in Kerala, we only kept those in three populated regions, i.e., Trivandrum HQ, Calicut, and Kochi. These robustness tests were conducted using event studies and included in the supplement, and the results are consistent with the baseline finding.

The fat tax was implemented by the newly elected Left Democratic Front (LDF) communist party. This tax predominantly affected branded restaurants that sold junk food, the majority of which were Western food chains. As a result, critics perceived the LDF's fat tax as politically motivated. We conducted a heterogeneous test to compare responses to the fat tax in pro-LDF and non-LDF districts using voting results from the 2016 Kerala general election. The results are provided in eFigure 2. Overall, we found that, the decreases in fast food purchases associated with the fat tax in pro-LDF districts and non-LDF districts were similar.

© 2023 Agarwal S et al. *JAMA Network Open*

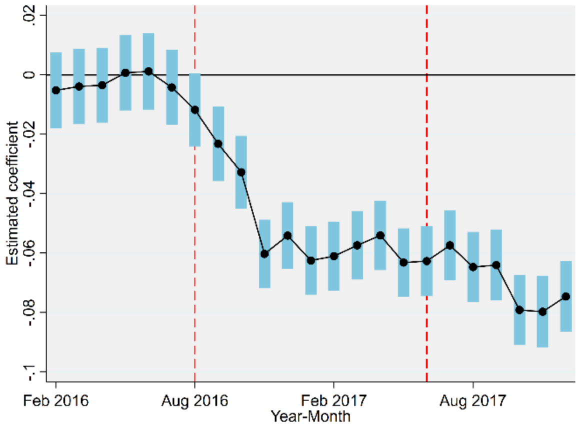

Panel A: Restricted accounts

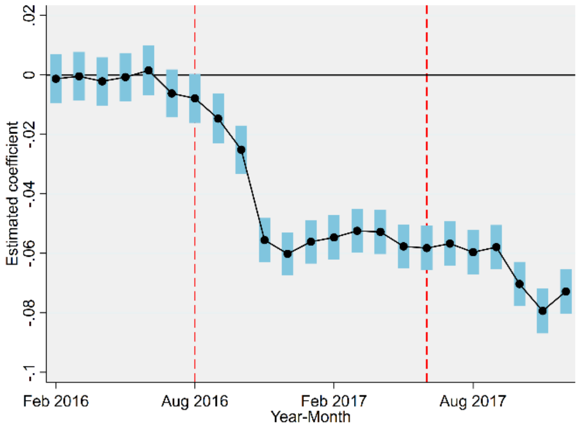

Panel B: Fast food frequency ratio

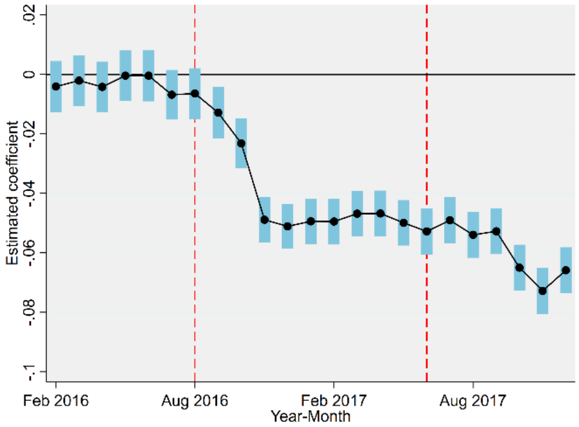

Panel C: Kerala populated regions vs other cities

eFigure 1. Robustness checks

This figure shows the dynamic changes in fast food purchase ratio using an event study design. In Panel A, we dropped accounts without any fast food purchases before August 2016. In Panel B, we replaced the outcome variable with the fast food frequency ratio. In Panel C, we compared three populated regions (Trivandrum HQ, Calicut, and Kochi) in Kerala with other major cities in India. Other specifications are the same as the baseline analysis in the main text. Two dotted vertical lines indicate the starting month (August 2016) and the ending month

© 2023 Agarwal S et al. *JAMA Network Open*

(June 2017) of the fat tax in Kerala. Shaded bars are the 95% confidence intervals. We controlled for account fixed effects and year-month fixed effects. Robustness standard errors are clustered at the account level.

© 2023 Agarwal S et al. *JAMA Network Open*

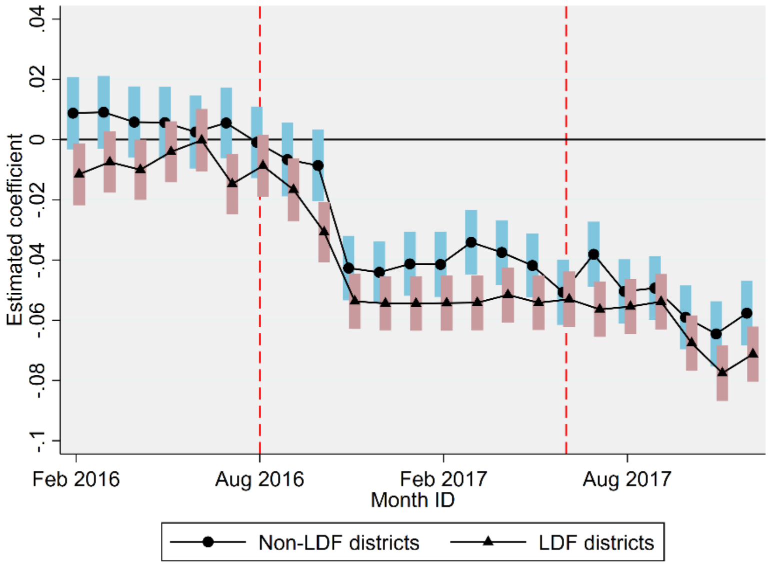

eFigure 2. Differential responses to the fat tax by political leaning

This figure shows the dynamic changes in fast food purchase ratio using an event study design. The outcome is the fast food ratio, defined as the proportion of fast food purchases in all food purchases. The treated group includes card accounts in Kerala, while the control group includes card accounts in other cities. The sample period is from 2016 to 2017. We showed differential responses to the fat tax between Kerala districts of different political leanings, i.e., pro-LDF districts and non-LDF districts. We split the sample period into 24 months taking January 2016 as the benchmark. Two dotted vertical lines indicate the starting month (August 2016) and the ending month (June 2017) of the fat tax in Kerala. Shaded bars are the 95% confidence intervals. We controlled for account fixed effects and year-month fixed effects. Robustness standard errors are clustered at the account level.

eTable. Summary statistics

Variable	Meaning	Full sample (238,015 accounts) Mean	SD	Kerala (87,303 accounts) Mean	SD	Other cities (150,712 accounts) Mean	SD
FastFoodRatio	fast food purchase amount as a proportion of all food purchase amount	0.170	0.323	0.164	0.315	0.174	0.326
Kerala	1 for Kerala, 0 for other cities	0.367	0.482	1.000	0.000	0.000	0.000
WithTax	1 for Aug 2016 – Jun 2017, otherwise, 0	0.458	0.498	0.458	0.498	0.458	0.498
AfterTax	1 for Jul 2017 – Dec 2017, otherwise, 0	0.250	0.433	0.250	0.433	0.250	0.433
Young	1 for age 18 – 30, 0 for age 31 – 90	0.370	0.483	0.745	0.436	0.104	0.305
Male	1 for men, 0 for women	0.805	0.396	0.827	0.378	0.789	0.408
Married	1 for married, otherwise, 0	0.586	0.493	0.425	0.494	0.725	0.446
PublicJob	1 for public job, otherwise, 0	0.050	0.219	0.066	0.248	0.037	0.190

This table shows the summary statistics of variables of the full sample, the exposed group (Kerala), and the control group (other cities).

© 2023 Agarwal S et al. *JAMA Network Open*
