# Supplementary material for: Association Between a State-Level Fat Tax and Fast Food Purchases
Source: JAMA Netw Open. 2023 Oct 16;6(10):e2337983. doi: 10.1001/jamanetworkopen.2023.37983 (PMC10580107; doi:10.1001/jamanetworkopen.2023.37983)
Supplement: Supplement 2. — Data Sharing Statement [file jamanetwopen-e2337983-s002.pdf]

Data Sharing Statement

Agarwal. Association Between a State-Level Fat Tax and Fast Food Purchases in India. *JAMA Netw Open*. Published October 16, 2023. doi:10.1001/jamanetworkopen.2023.37983

Data
Data available: No
